# A novel multisite model to facilitate hepatitis C virus elimination in people experiencing homelessness

**DOI:** 10.1016/j.jhepr.2024.101183

**Published:** 2024-08-12

**Authors:** Adele Mourad, Rona McGeer, Emma Gray, Anna-Marie Bibby-Jones, Heather Gage, Lidia Salvaggio, Vikki Charles, Natasha Sanderson, Margaret O’Sullivan, Thomas Bird, Sumita Verma

**Affiliations:** 1 Department of Gastroenterology and Hepatology, University Hospitals Sussex NHS Foundation Trust, Brighton, UK; 2 Department of Clinical and Experimental Medicine, Brighton and Sussex Medical School, Brighton, UK; 3 Sussex Partnership NHS Foundation Trust, Brighton, UK; 4 Department of Clinical and Experimental Medicine, University of Surrey, Guilford, UK; 5 School of Biosciences, Faculty of Health and Medical Sciences, Royal Surrey NHS Foundation Trust, Guildford, UK

**Keywords:** Homeless persons, Health-related quality of life, Health care economics, Community services, Re-infection, Drug overdose

## Abstract

**Background & Aims:**

Only a handful of countries are on target to achieve elimination of HCV by 2030. People experiencing homelessness (PEH) remain an important HCV reservoir. The END C study evaluated clinical, patient reported, and health economic outcomes of a decentralised integrated model.

**Methods:**

This prospective study assessed a decentralised regional service based at multiple homeless sites in southeast England. Novel linkage-care strategies were used. We assessed generic and liver specific health-related quality of life (HRQoL) (SF-12v2; EQ-5D-5L, and SFLDQol) pre-/post-HCV treatment, and cost per HCV case detected and cured. The primary outcome was sustained virological response (SVR12) in the intention-to-treat (ITT) population.

**Results:**

We recruited 418 individuals with mean age 44.45 ± 10.6 years, 78% were male, 74% were currently homeless, current injecting drug use or alcohol use was 25% and 65%, respectively. Prevalence of cirrhosis (liver stiffness measurement ≥12 kPa) was 12%. A total of 28% (n = 116) were HCV PCR-positive of whom 105 individuals received direct acting antiviral treatment. The ITT SVR12 rates were 81% (95% CI 72%–88%), the only predictor of SVR12 was >80% treatment adherence (OR 20.69, 95% CI 6.227–68.772, *p <*0.001). HRQoL improved significantly after SVR12: SF-12v2 (General Health, Mental Health, Social Functioning, Mental Health Composite Score *p <*0.049); SFLDQoL (Symptoms/Effects of Liver Disease, Distress, Loneliness *p <*0.004) and EQ-5D-5L (Index Score, Visual Analog Scale *p <*0.001). Costs (British pound 2022) per HCV case detected and per case cured were £359 and £257, respectively. Reinfection rates were 6.82/100 person years.

**Conclusion:**

The END C study endorses a multisite decentralised service for PEH enabling excellent linkage to care, high SVR12 rates, and significant improvements in generic and liver specific HRQoL, all being achieved at modest costs. Such services are paramount to help achieve HCV elimination.

**Impact and implications:**

In people experiencing homeless, we found a high prevalence of HCV, alcohol, and substance misuse including overdoses and mental health issues. Despite this, an integrated and decentralised service resulted in excellent linkage to care with high SVR12 rates. Even in this complex cohort with multiple comorbidities, SVR12 was associated with significant improvements in both generic and liver specific HRQoL. This was all achieved at modest costs in a community setting. Such models of care are feasible, easy to replicate, and essential if we are to achieve HCV elimination.

## Highlights

• People experiencing homelessness (PEH) have a high prevalence of HCV and remain an important reservoir for HCV.
• A multisite regional decentralised service is effective in linking PEH into care with high SVR12 rates.
• Achieving SVR12 results in significant improvements in generic and liver-specific health-related quality of life in PEH.
• HCV detection and cure can be achieved at modest costs in a community setting.

## Graphical abstract

**Figure:**
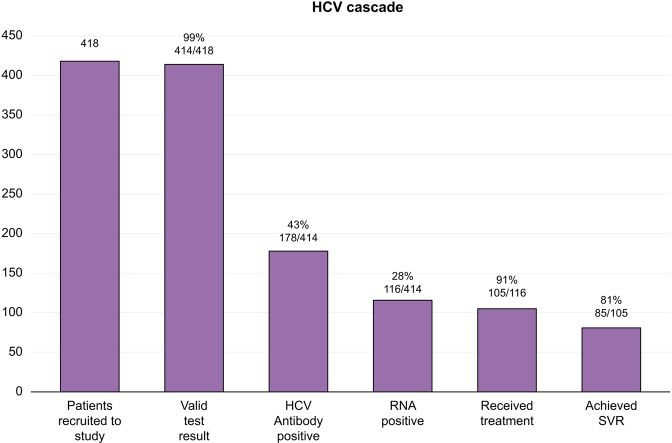

## Introduction

People experiencing homelessness (PEH) continue to have significant health disparities compared with those living in stable housing. This includes high prevalence of alcohol and substance use disorder, with an increased risk of acquiring blood borne viruses (BBV) and other infectious diseases.^1^ A recent systematic review reported alcohol and recent injecting drug use (IDU) in 59% and 21% of PEH, respectively, with a 20% HCV prevalence.^2^ Globally, 58 million individuals have HCV infection with only one in four diagnosed and less than 15% treated.^3^ In England, in 2023, approximately 62,600 individuals still lived with chronic HCV infection,^4^ almost all being people who use drugs (PWUD).^5^ Approximately 50% of PWUD report homelessness.^6^

PEH remains an important reservoir for HCV infection with high drop offs in the HCV care cascade including screening, linkage to care, and treatment.^7^ Even if referred for HCV treatment, PEH are less likely to receive HCV therapy^8^ with as low as 4% completing treatment.^7^ Potential reasons could include stigmatisation and inability of traditional hospital-based models to engage PEH. Conversely, decentralised, integrated care is associated with high levels of satisfaction, compliance, and cure rates ranging from 82%–92%.^2^^,^^6^^,^[9], [10], [11], [12]

Although prior studies have assessed decentralised models in PEH,^2^ the focus has been on clinical outcomes. There is a paucity of published literature assessing the impact of sustained virological response (SVR) on health-related quality of life (HRQoL) in PEH. Additionally, very few studies have looked at the costs of HCV detection and cure amongst PEH.

The main objectives of the END C study were to establish and evaluate a novel community service for PEH. Besides clinical outcomes, we also aimed to assess patient reported and health economic outcomes following a sustained virological response (SVR12) in PEH.

## Patients and methods

### Details of the care model

In England, specific direct acting anti-viral (DAA) regimen is determined and funded by the National Health Service England (NHSE). This is delivered by 22 national centres (Operational Delivery Networks-ODNs).^13^ This study was set in the Sussex ODN on the southeast coast of England.

We have expertise in establishing novel community models of care in addiction centres and community homeless sites. Based on our earlier work^6^^,^[9], [10], [11]^,^^14^ and links with various stakeholders (addiction centres, homeless shelters, charities, primary care physicians, and peer mentors), we established the END C study. This 4-year regional study (Aug 2019 to July 2023, extended by 2-years as a result of the COVID-19 pandemic), was based at multiple homeless shelters and community centres in the Sussex ODN (Fig. S1). All clients attending these homeless sites were approached by the research fellow/hepatitis nurse/peer mentor, provided with a short patient information sheet, and if the individual was willing, gave informed consent. The aim on the first visit was to offer BBV testing, transient elastography (TE), peer support, and to complete baseline data collection. BBV testing was done using either venous blood, or finger prick testing (dry blood spot testing [DBST] or capillary blood testing [CBT]).

#### Venous blood samples

Testing included HCV antibody, HBsAg, hepatitis B core antibody (HBcAb) and HIV antibody. If HCV antibody was positive, quantitative HCV polymerase chain reaction (PCR) analysis was done by reflex using the Abbott Real-Time HCV RT-PCR, with the lower limit of detection (LLD) being 12 IU/ml. HCV genotype was requested additionally at a second visit (Micropathology Laboratory, Coventry, UK (LLD ∼1,000 IU/ml).

#### Finger prick capillary blood test

The CBT was performed at St George’s Hospital Southwest London Pathology and included HCV antibody, HBsAg, HBcAb, and HIV antibody. Quantitative HCV PCR and genotype were performed by reflex. LLD for HCV RNA was ∼2,000 IU/ml.

#### Finger prick dry blood spot testing

DBST was performed at Manchester Public Health England and included HCV antibody, HBsAg, HBcAb, and HIV antibody. HCV qualitative and quantitative PCR were performed by reflex. LLD for HCV RNA was 660 IU/ml. Genotyping was done by CBT.

For all the above tests, HCV PCR results were usually available within 2 weeks of testing.

The DAA regimen was determined nationally.^13^ All individuals with a positive HCV PCR were discussed at the weekly regional multidisciplinary meeting (MDM) with a national requirement to genotype at least 75% of those treated. These requirements have been relaxed recently. We aimed to commence DAA treatment within 3 to 4 weeks of HCV diagnosis. Following commencement of DAAs, individuals were monitored as clinically indicated either via phone or in person. Individuals with cirrhosis were referred to their local hospitals for hepatocellular cancer (HCC) and variceal surveillance.

Novel aspects of our model^6^^,^^9^^,^^10^ were:

• Decentralised service, *i.e.* based in the community and not in secondary or tertiary care settings. Additionally, we adopted a non-judgemental and personalised approach, and ongoing alcohol and IDU were not barriers to receiving HCV treatment.
• ‘One-stop’ service, *i.e.* aimed to provide as many elements as possible of the service on the first visit.
• Easy access to staff (mobile phone contact).
• Flexibility in how BBV testing was provided to include venous and finger prick testing.
• Onsite provision of TE.
• Novel DAA dispensing including home delivery and installing lockers in homeless sites. To reduce clinic visits, where possible, we dispensed the entire DAA course on a single occasion.
• Minimal monitoring—only complex cases (*e.g.* those with cirrhosis) underwent blood monitoring. We restricted blood tests to initial BBV testing and then assessment of SVR12.
• Test and treat strategy with an aim to commence DAAs within 3 to 4 weeks of HCV diagnosis.
• Use of peer mentors. Our two peer mentors were individuals with lived experience of HCV and were trained and funded by the Hepatitis C Trust, the largest HCV charity in the UK. They attended the community clinics along with the research fellow/hepatitis nurse, using their lived experience to encourage clients to overcome barriers to BBV testing and HCV treatment. If required, they accompanied clients to the community clinic during DAA treatment follow up and during hospital visits for HCC and variceal surveillance. They remained in telephone contact with clients, reminded them about clinic appointments and monitored their progress and medication adherence. Finally, they liaised with other agencies including addiction centres and outreach support teams.
• Contingency management. We provided £10 food vouchers at the time of BBV testing, initiating DAA treatment and attending for SVR12 blood sampling.
• Assessment of HCV reinfection 6–12 months after achieving SVR12.

Further details on setting up a community HCV service can be found in an earlier publication.^14^

### Inclusion/exclusion criteria

All adults attending the homeless sites and who were willing and able to give informed consent were included. Those unwilling to provide informed consent were still offered the service, but their data were not collected.

### Study definitions

Homeless was defined as street homeless and/or living in temporary accommodation at the initial assessment. PWUD included those with current or history past drug use (injecting or non-injecting) and/or those currently receiving opioid agonist treatment (OAT). Current injecting or non-injecting drug use or alcohol use was defined as use within the past 6 months. Suitability for HCV treatment consisted of willingness and motivation to engage with HCV treatment. SVR12 was defined as the absence of detectable virus 12 weeks after end of treatment. Reinfection was determined by any level of detectable virus 6–12 months after achievement of SVR12. Cirrhosis (METAVIR F4) was defined as liver stiffness measurement (LSM) ≥12 kPa.^15^^,^^16^ The ITT analysis included all individuals commencing HCV treatment. The modified ITT (mITT) analysis excluded individuals who did not attend SVR12 blood sampling.

### Data collection

All data, including clinical, were prospectively collected onto an anonymised database.

### Patient reported outcome measures (PROMs)

All individuals commencing HCV treatment were offered HRQoL assessment prior to (pre-treatment) and at end of DAA (post-treatment). Liver specific HRQoL was assessed using the Short Form Liver Disease Quality of Life Questionnaire (SFLDQoL);^17^ generic HRQoL was assessed using the Short-Form-12 health survey v2 (SF-2v2) and EQ-5D-5L questionnaires.^18^^,^^19^ The SFLDQoL has nine domains (Distress, Stigma, Memory, Symptoms of Liver Disease, Sleep, Hopelessness, Effects of liver disease, Loneliness, and Sexual Function; scale of 0–100, higher score indicates better QoL).^17^ SF-12 v2 has 10 domains (Role Physical, General Health, Vitality, Physical Functioning, Role Emotional, Social Functioning, Bodily Pain, Mental Health, Physical Health Composite (PHCS) score, and Mental Health Composite Score (MHCS); scale of 0-100, higher score indicates better health).^18^ The EQ-5D-5L has a five-item Composite Profile Score scored on a five-point scale and converted to an index value range (0.57–1.00) and a 20 cm vertical Visual Analogue Scale (range 0 [worst] to 100 [best]).^19^ File S1 shows how the questionnaires were analysed and scored.

### Health economic data

Data were collected from consenting individuals undergoing BBV screening and DAA treatment. All steps in an individual’s care pathway were recorded by the research fellow on an excel spreadsheet in a micro costing exercise. This included the initial 30-min consultation and BBV screening and, where relevant, subsequent tests, contacts with the research fellow and peer mentors (in minutes) during follow up assessment and included SVR12 outcomes.

### Study outcomes

The primary outcome was SVR12 (intention-to-treat [ITT]). Secondary outcomes included HCV prevalence, HCV treatment outcomes, DAA adherence, HCV reinfection, changes, if any, in generic and liver specific HRQoL post HCV treatment, and cost per HCV case detected and cost per HCV case cured.

### Data analysis

#### Clinical data

Based on our prior work^6^^,^^9^^,^^10^ we aimed to recruit approximately 400 individuals with an estimated 30% being HCV PCR-positive and approximately 100 individuals receiving DAA treatment. Data are summarised using counts, means + SD, medians (IQR), or percentages and Student’s t-test and Chi-Square test for continuous and categorical variable respectively. Logistic regression analysis was used to model the relationship between the binary dependent outcomes (0 *vs.*1), cirrhosis (yes/no), HCV RNA positive (yes/no), treatment outcome (SVR12 yes/no) and key independent factors (age, sex, current IDU, IDU current/past, current non-IDU, shared paraphernalia ever, alcohol use (current/past), current alcohol use, receiving OAT, history of overdose, history of incarceration, homeless at initial assessment, any psychiatric diagnosis, contact with peer mentor, fibrosis stage, HCV treatment regimen and duration and adherence). A multivariate logistic regression model was then derived to look at the relationship between the key factors and the dependent outcome. To build the model, the statistically significant key factors from bivariate analysis were added to the null model using forward selection, where the factor with the highest significant *p* value (*p <*0.05), based on the likelihood ratio test, was added next. Factors were removed from the model if *p* ≥0.05. Only statistically significant variables on multivariate analysis are reported.

HCV reinfection rate was calculated in those achieving SVR12 as the number of reinfections observed in the study period divided by the sum of all the years each individual was observed for, multiplied by 100 (per 100 person years).

### Statistical analysis of patient reported outcomes

Differences in pre- and post-HCV treatment scores were calculated (mean ± standard deviation) and compared using paired Student's t-test. Because the PROMs sample size of was small (see below) robust data completion was not possible to build imputation models using this data. Bootstrapping (with 1,000 reps) was used to address missing data for PROMs with <50% missing. A sensitivity analysis was conducted by establishing whether the conclusions from the bootstrapped analysis differed to those from a complete case analysis. For all statistical analysis *p* <0.05 was considered significant. Statistical software used was STATA v.18.^20^

### Health economic analysis

To reflect real world experience, service provision by the research fellow (time spent in consultation with PEH for screening and treatment) was costed as for a Band 7 nurse. We used nationally validated rates (at £66/h including oncosts and NHS facility overheads, *i.e.* £1.10/min, British pound 2022).^21^ Costs of tests were obtained from a local NHS laboratory. Cost per case detected was calculated as the total cost of all nurse time and tests performed, summed across all those invited to be screened, divided by the total number of individuals with a positive HCV RNA (a case), over the study period. Cost per screen was the total cost of all screens conducted divided by the number of people screened. To calculate the cost per cure of those receiving DAA, the total cost of tests and nurse time was summed across all individuals receiving treatment and divided by the total number of individuals achieving SVR12. Sensitivity analyses explored the impact of screening method on cost per case detected and of varying the cost of the fibroscan on cost per case cured. DAA costs and time spent in presenting cases at the weekly regional MDM were not included. Peer mentor input at the first visit and during treatment were excluded from the analysis as data were incompletely recorded.

## Results

### Clinical outcomes

**Fig. 1:**
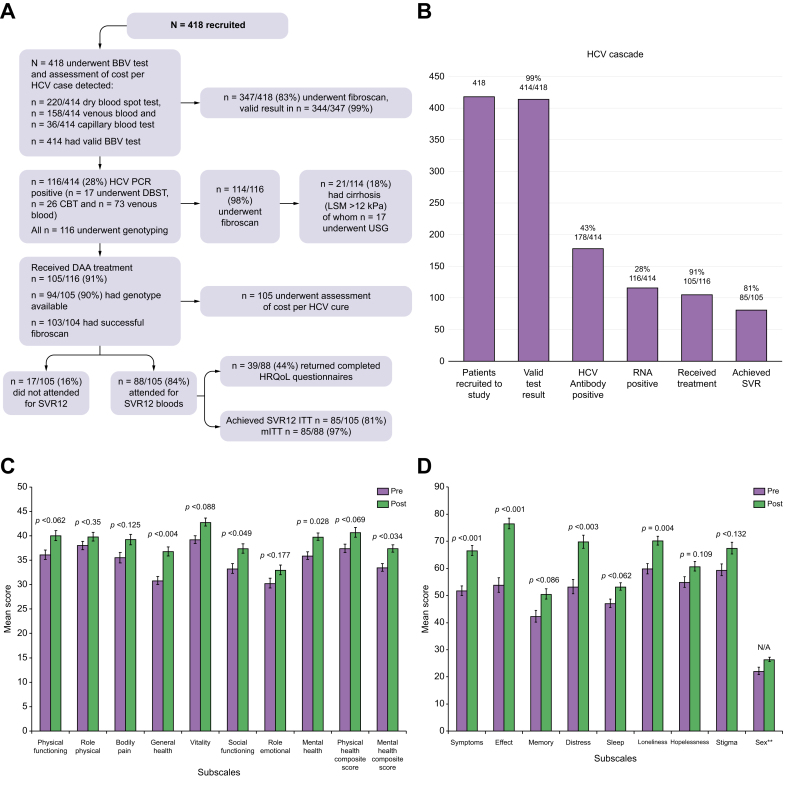
Participant flow chart and care cascade. (A) Participant flow chart, (B) HCV care cascade, (C) Mean (with SE bars) SF-12 scores pre and at end of HCV treatment in those achieving SVR12 (n = 39); (D) Mean (with SE bars) SFLDQoL scores pre and at end of HCV treatment in those achieving SVR12 (n = 39).

A total of 418 individuals were recruited, mean age 44 ± 10.6 years, 78% being male (Table 1 and Fig. 1a). All individuals had a history of homelessness, 74% being homeless at initial assessment. Prevalence of current IDU and alcohol use were 25% (95% CI 21%–29%) and 65% (95% CI 60%–69%), respectively. Forty-seven percent of the cohort had a history of incarceration with 38% having a history of taking an overdose. There was high prevalence of mental health comorbidity. About 60% had had a prior test for HCV (Table 1). Twenty-eight percent (95% CI 24%–33%) (n = 116) were HCV PCR-positive, of whom n = 27 (26%) were new diagnoses. Successful genotyping was available in 104/116 (90%) (genotype 3, n = 64, 62%; genotype 1a, n = 36, 35%). Predictors of a positive HCV PCR were IDU (current/past) OR 14.46, 95% CI 5.75–36.39, *p <*0.001; OAT OR 2.39, 95% CI 1.35–4.23, *p* = 0.003; homelessness OR 0.45, 95% CI 0.23-0.86, *p* = 0.015; and shared paraphernalia OR 2.20, 95% CI 1.18-4.09, *p* = 0.013 (Table S1). Of the 344 individuals with a valid TE result, prevalence of cirrhosis (LSM ≥12 kPa) was 12%. Independent predictors of cirrhosis were HCV PCR positivity OR 4.02, 95% CI 1.89–8.57, *p <*0.001 and currently drinking alcohol OR 1.01, 95% CI 1.00–1.01, *p <*0.001 (Table S2). Thirty-nine individuals had received prior HCV treatment (Table 1).

**Table 1:** Baseline demographic and clinical data (n = 418) (in 4 individuals, the BBV test was invalid).

	Whole cohort (N = 418)	HCV PCR positive (n = 116)	HCV PCR negative (n = 298)	*p* value
Age, years	44.45 ± 10.60	43.64 ± 9.63	44.82 ± 10.97	0.289
≥60	37 (9%)			
<60	381(91%)			
Male sex	324 (78%)	90 (78%)	230 (77%)	0.930
Prior history of homelessness	418 (100%)			
Currently homeless	310 (74%)	80 (69%)	229 (77%)	0.098
Ever incarcerated	195 (47%)	74 (64%)	119 (40%)	*<*0.001
Alcohol use ever	357 (85%)	90 (78%)	263 (88%)	0.006
Current alcohol use (in past 6 months)	270 (65%)	61 (53%)	205 (69%)	0.002
Non-IDU ever	328 (79%)	104 (90%)	220 (74%)	*<*0.001
Current non-IDU (in past 6 months)	254 (61%)	87 (75%)	165 (55%)	*<*0.001
Non IDU frequency:				
Daily	94/254 (37%)	36/87 (41%)	57/165 (36%)	
2/3 times a week	60/254 (24%)	21/87 (24%)	38/165 (23%)	
Weekly	69/254 (27%)	24/87 (28%)	45/165 (27%)	
Monthly	31/254 (12%)	6/87 (7%)	25/165 (15%)	
IDU ever	208 (50%)	109 (94%)	99 (33%)	*<*0.001
Current IDU (in past 6 months)	103 (25%)	59 (51%)	44 (15%)	*<*0.001
IDU frequency in past 6 months				
Daily	49/103 (48%)	33/59 (56%)	16/44 (36%)	
2/3 times a week	19/103 (18%)	11/59 (19%)	8/44 (18%)	
Weekly	15/103 (15%)	6/59 (10%)	9/44 (20%)	
Monthly	20/103 (19%)	9/59 (15%)	11/44 (25%)	
Shared paraphernalia ever	172 (41%)	91 (78%)	81 (27%)	*<*0.001
Drug overdose ever	160 (38%)	63 (54%)	96 (32%)	*<*0.001
On OAT	129 (38%)	76 (66%)	52 (17%)	*<*0.001
Support network (self-reported)				
Poor	84 (20%)	27 (23%)	57 (19%)	0.346
Minimal	137 (33%)	37 (32%)	100 (34%)	0.747
Good	197 (47%)	52 (45%)	141 (47%)	0.649
Physical Comorbidity	249 (60%)	85 (73%)	163 (55%)	*<*0.001
Mental health issues∗	319 (76%)	99 (85%)	217 (73%)	
Depression	248 (59%)	83 (72%)	163 (55%)	0.002
Anxiety	197 (47%)	59 (51%)	136 (46%)	0.339
PTSD	54 (13%)	17 (15%	37 (12%)	0.544
Other	94 (22%)	32 (28%)	59 (20%)	
Ever had prior HCV test	247 (59%)	102 (88%)	143 (48%)	
Prior HCV treatment	39	14	25	
Nature of prior HCV treatment	27 DAA, 11IFN, 1 unknown	8 DAA, 6 IFN	19 DAA, 5 IFN, 1 unknown	
HCV antibody positive	178 (43%)	116	62	
HCV PCR positive	116/414 (28%)			
Underwent TE	347 (83%)			
Valid TE	344/347 (82%)			
				
∗∗F0-F1 (<7.1 kPa)	256 (74%)	72/113 (64%)	180/227 (79%)	0.002
∗∗F2-F3 (7.1 kPa-11.9 kPa)	47(14%)	20/113 (18%)	27/227 (12%)	0.144
∗∗F4 (≥12 kPa)	41 (12%)	21/113 (19%)	20/227 (9%)	0.009

BBV blood borne virus; IDU injecting drug use; DAA direct acting antivirals; IDU, injecting drug use; IFN, interferon; TE transient elastography; OAT opioid agonist treatment; PTSD post-traumatic stress disorder.

Data are summarised using counts, means ± SD, medians (IQR), or percentages. Student’s *t* test and Chi-Square test were utilised for analysis of continuous and categorical variable respectively.

∗ Most had more than one mental health issue.

∗∗ Refs.^15^,^16^.

### HCV treatment outcomes

Of those with a positive HCV PCR (n = 116), DAA treatment was commenced in 105 (91%) (Fig 1A and 1B), the remainder were not willing to engage further with HCV treatment. Table 2 shows the clinical and treatment outcomes. Eighteen percent (19/103) of the treated cohort had cirrhosis (LSM ≥12 kPa). Sofosbuvir-based DAA regimens were the most commonly used (66/105, 63%). ITT SVR12 were 85/105 (81%) (95% CI 72%–88%) (Fig. 1a). Of the 20 non-SVRs, there were three virological failures: two non-responders (successfully retreated with sofosbuvir/velpatasvir/voxilaprevir) with one being a responder relapser. All three self-reported ≥80% DAA adherence and were treatment naive. The remaining 17 individuals did not attend for SVR12 blood sampling: lost to follow up (LTFU) (n = 9), poor compliance (n = 6), and death (n = 2) (Table 2). Modified ITT SVR12 rates therefore were 85/88 (97%) (95% CI 90%–99%). The only predictor of SVR12 was ≥80% treatment adherence (OR 20.69, 95% CI 6.227–68.772, *p <*0.001) (Table 3). Of those with ≥80% adherence, 92% (78/85) achieved SVR12 *vs.* 7/20 (35%) with <80% adherence, *p <*0.001. Fig 1b shows the HCV care cascade.

**Table 2:** Clinical and treatment data in individuals who received direct acting antivirals (n = 105).

DAA regimen	Sofosbuvir/ledipasvir	Sofosbuvir/velpatasvir/voxilaprevir	Elbasvir/grazoprevir	Sofosbuvir/velpastasvir ± ribavirin	Glecaprevir/pibrentasvir
Number	9 (9%)	2 (1%)	20 (19%)	55 (52%)	19 (18%)
Age, years	40.67 ± 7.68	46.5 ± 10.60	47 ± 11.88	42.93 ± 9.47	47.47 ± 8.78
Male sex	8 (89%)	1 (50%)	18 (90%)	39 (71%)	15 (79%)
Comorbidity	6 (67%)	1 (50%)	17 (85%)	39 (71%)	13 (68%)
IDU ever	9 (100%)	2 (100%	18 (90%)	53 (96%)	17 (89%)
IDU current	6 (67%)	1 (50%)	9 (45%)	27 (49%)	9 (47%)
Current non-IDU	7 (78%)	1 (50%)	17 (85%)	42 (76%)	12 (63%)
Alcohol ever	8 (89%)	2 (100%	14 (70%)	44 (80%)	15 (79%)
Current alcohol	4 (44%)	1 (50%)	9 (45%)	31 (56%)	12 (63%)
Any psychiatric diagnosis	9 (100%)	2 (100%)	18 (90%)	45 (82%)	18 (95%)
On OAT	7 (78%)	1 (50%)	12 (60%)	39 (71%)	11 (58%)
Ever been to prison	7 (78%)	1 (50%)	14 (70%)	34 (62%)	9 (47%)
Genotype 1a	9 (100%)	0	20 (100%)	0	3 (16%)
Genotype 2	0	0	0	0	2 (11%)
Genotype 3	0	2 (100%)	0	49 (89%)	9 (47%)
No genotype available	0	0	0	6 (11%)	3 (16%)
Liver tests (n = 70)					
Platelet x10^9^/L (± SD)	207 ± 129	236 ± 7.1	276 ± 91	207 ± 99	274 ± 75
ALT IU/L (IQR)	32 (24)	72 (6)	65 (107)	85 (98)	53 (41)
Bilirubin (μmol/L) (IQR)	10 (10)	9 (2)	9 (8)	8 (10)	6 (5)
Albumin (g/L) (IQR)	37 (15)	43 (0)	43 (5)	43 (7)	43 (3)
INR (IQR)	1.1 ± 0.1	1 ± 0	1 ± 0.1	1.1 ± 0.2	1 ± 0.1
LSM∗					
F0-F1 (<7.1 kPa)	8 (89%)	1 (50%)	14 (70%)	31 (58%)	11 (58%)
F2-F3 (>7.1–11.9 kPa)	1 (11%)	0	5 (20%)	9 (17%)	4 (21%)
F4 (≥12 kPa)	0	1 (50%)	1 (5%)	13 (25%)	4 (21%)
Received prior treatment and nature of treatment	n = 1 DAA	n = 2 DAA	n = 1 DAA, n = 2 IFN	n = 3 DAA, n = 3 IFN	n = 1 DAA, n = 1 IFN
Treatment duration					
8 weeks	9 (100%)	0	0	0	17 (89%)
12 weeks	0	2 (100%)	20 (100%)	55 (100%)	2 (11%)
≥80% treatment adherence	6 (67%)	2 (100%)	16 (80%)	46 (84%)	15 (79%)
SVR12	6 (67%)	2 (100%)	16 (80%)	45 (82%)	16 (84%)
Reasons for non-SVR	LTFU n = 2 Poor compliance n = 1	NA	Poor compliance n = 3 LTFU n = 1	LTFU n = 6 Died n = 2 RR n = 1 NR n = 1	Poor compliance n = 2 NR n = 1

IDU, injecting drug use; IFN, interferon; LSM, liver stiffness measurement; LTFU lost to follow up; NR non responder; OAT, opioid agonist treatment; RR responder relapse; SVR12, sustained virological response.

Normal values bilirubin 21 μmol/L, ALT 0-41 IU/L, albumin 35–52 g/L, INR 0.8–1.2, platelets 150–450 × 10^9^/L. Data are summarised using counts, means ± SD, medians (IQR), or percentages.

∗ n = 104 underwent TE, successful in n = 103.

**Table 3:** Univariate and multivariate analysis of predictors of SVR12.

Key variables	Univariate analysis			Multivariate analysis		
	OR	95%	*p* value	OR	95%	*p* value
Age (per year increase in age)	0.97	0.924–1.023	0.279			
Male	0.54	0.143–2.186	0.358			
IDU (current/past)	2.25	0.382–13.24	0.370			
Current IDU	2.09	0.759–5.752	0.154			
Current non-IDU	1.39		0.548			
Alcohol use (current/past)	2.5	0.858–7.361	0.093			
Currently drinking alcohol	1.24	0.466–3.280	0.669			
Receiving OAT	1.43	0.525–3.91	0.483			
Drug overdose	1.59	0.595–4.224	0.356			
Ever incarcerated	1.83	0.686–4.90	0.227			
Homeless at initial assessment	0.97	0.336–2.812	0.959			
Contact with peer mentor	1.36	0.606–3.626	0.545			
Any psychiatric diagnosis	0.75	0.152–3.674	0.720			
Cirrhosis (F4 *vs.* F0–F3)	0.31	0.101–0.920	0.035			
Genotype 3 *vs.* non 3	1.12	0.418–2.968	0.830			
Treatment sofosbuvir *vs.* non sofosbuvir	1.13	0.425–2.981	0.813			
8 weeks *vs.* >8 weeks	0.72	0.244–2.114	0.548			
≥80% treatment adherence	20.69	6.227–68.772	<0.001	20.69	6.227–68.772	<0.001

IDU, injecting drug use; OAT opioid agonist treatment; SVR12, sustained virological response.

Logistic regression analysis was used to model the relationship between the binary dependent outcomes (0 *vs.*1), (SVR12 yes/no) and key independent factors (see section on statistical analysis). A multivariate logistic regression model was then derived to look at the relationship between the key factors and the dependent outcome. To build the model, the statistically significant key factors from bivariate analysis were added to the null model using forward selection, where the factor with the highest significant *p* value (*p <*0.05), based on the likelihood ratio test, was added next. Factors were removed from the model if *p* ≥0.05. Only statistically significant variables on multivariate analysis are reported.

### Reinfection data

Of the 78/85 individuals who achieved SVR12 and survived, 47 (60%) were retested for HCV reinfection 6–12 months post SVR12. Of these 47 individuals, three (6%) developed reinfection (6.82/100 person years).

### Mortality

Of the whole cohort (N = 418), 38 (9%) individuals died. Causes of death were identified through health records and were available for 29 individuals. This included drug overdose n = 11, liver related n = 5 (decompensated cirrhosis n = 4 and HCC n = 1), and other causes n = 13. Of the treated cohort (n = 105), n = 10 (10%) died, with eight having a known cause of death: four resulting from drug overdose, two mental health related, one related to liver disease and one to head injury.

### Patient reported outcomes

Of the 88 individuals attending for SVR12 bloods, completed pre- and post-questionnaires were available in 39 (44%), all achieved SVR12. In 72%, sexual function domain of SFLDQoL questionnaire was missing and therefore were excluded from analysis. Fig 1C and 1D show the mean changes in SF-12v2 and SFLDQoL scores respectively, pre- and post-HCV treatment in the 39 individuals who achieved SVR12. Raw scores are shown in Table S3. There were significant improvements (*p <*0.049) in the following SF-12v2 domains: General Health, Mental Health, Social Functioning, and Mental Health Composite Score (Fig 1C). The SFLDQoL questionnaires showed significant improvements in the following domains: Symptoms of Liver Disease, Effects of Liver Disease, Distress and Loneliness (*p <*0.004) (Fig. 1d). Compared with baseline, there were also significant improvements (*p <*0.001) in the EQ-5D-5L scores at the end of HCV treatment: Index Score 0.40 ± 0.30 *vs.* 0.50 ± 0.30 and Visual Analog Scale 44.9 ± 21 *vs.* 56.5 ± 17.5.

### Health economic outcomes

Based on 30 min for the initial consultation (£33), the cost per HCV case detected was £359, the cost per screen being approximately £100 (Table 4). The unit costs of tests used in the calculations are shown in Table S4. The cost per case detected was dependent on the choice of screening tests used. If only DBST and CBT were used (replacing venous blood testing with CBT), the cost per case detected was £282 and the cost per screen dropped to £78 (Table S5).

**Table 4:** Cost per HCV case detected (n = 418).

Test type	Total screened	Screening outcomes	n	Cost of screening (£)	Total cost (£)∗
DBST	223	Initial screen only (HCV antibody negative)-includes 3 failed screens	167	21.42	3,577
		Follow up test if HCV antibody positive but HCV PCR-negative	39	82.91	3,233
		Follow up test for genotype if HCV PCR-positive (performed using CBT)	17	102.91	1,749
		Total	223		8,559
CBT	36	Initial screen only (HCV C antibody-negative)	1	16.57	17
		Follow up test if HCV antibody-positive but HCV PCR-negative	9	58.85	530
		Follow up test for genotype if HCV PCR-positive	26	78.85	2,050
		Total	36		2,597
Venous	159	Initial screen only (HCV C antibody-negative), includes 1 failed	72	34.83	2,508
		Follow up test if HCV antibody-positive but HCV PCR-negative	14	104.23	1,459
		Follow up test for genotype if PCR-positive	73	173.75	12,684
		Total	159		16,651
All	418	Total of DBST, CBT and venous blood tests∗∗	418		27,807
		+ Cost of nurse initial 30-min consultation: £33 per participant∗∗∗	418	33	13,794
		GRAND TOTAL OF SCREENING COSTS	418		41,601

CBT, capillary blood test; DBST, dry blood spot testing.

Cost per case detected was calculated as the total cost of all nurse time and tests performed, summed across all those invited to be screened, divided by the total number of individuals with a positive HCV RNA (a case), over the study period. Cost per screen was the total cost of all screens conducted divided by the number of people screened.

Number of cases detected: N = 116 (n = 17 by BST; n = 26 by CBT; n = 73 by venous blood).

Cost per case detected: 41,601/116 = £359.

Cost per screen: 41,601/418 = £100.

∗ Total costs are rounded to the closest £.

∗∗ Test costs are in Table S4.

∗∗∗ Cost per hour, including oncosts and NHS facilities overheads of a Band 7 Nurse is £66. Ref.^21^.

Participants undergoing treatment were contacted by the research fellow as needed, either face-to-face or by telephone. The mean number of minutes of contact between the research fellow and participants was 129 (range 50–395 min) representing a mean of 5.1 face-to-face contacts and 3.6 phone contacts. The cost per HCV case cured was £257 (Table 5), excluding DAA and peer mentor costs. Given the uncertainty in the cost of the fibroscan (see Table S4), a second calculation was undertaken at a cost of £100 per scan (rather than £50), resulting in a cost per HCV cure of £318 (Table S6).

**Table 5:** Cost per case cured (n = 105).

Item	Number receiving	Unit cost (£)	Total cost (£)∗
Fibroscan∗∗	104	50	5,200
Abdominal ultrasound∗∗	17	61	1,037
Contact with hepatology nurse Band 7; mean duration of contact = 129 min, at £1.10/min∗∗∗	105	129 min at £66 per h = £142	14,900
SVR12 blood by DBST	88	7.91	696
Total cost			21,883
Cost per person achieving SVR12 = 21,883/85 = £257			

DBST, dry blood spot test; SVR12, sustained virological response.

To calculate the cost per cure of those receiving DAA, the total cost of tests and nurse time was summed across all individuals receiving treatment and divided by the total number of individuals achieving SVR12.

∗ Total costs are rounded to the closest £

∗∗ Test costs are in Table S4.

∗∗∗ Cost per hour, including oncosts and NHS facilities overheads of a Band 7 Nurse is £66. Ref.^21^.

Although 64 individuals received peer support, the time spent was only recorded for n = 30 of them with an average of 260 min per person (range 70–660 min). Since the extent of peer support was incompletely recorded it was not included in the main analysis. Applying the weekday hourly cost of a home care worker (£23).^21^ to the available data, yielded a cost of approximately £100 per person. Peer mentors in the study were reimbursed through charity funding.

## Discussion

Our regional multisite decentralised service for PEH was successful in linking one of the most vulnerable and disenfranchised cohorts into care. This model was based on our earlier single site work^6^^,^^9^^,^^10^ and in the current study we showed that such a service can be replicated regionally. We found high disease burden from liver disease in this population with an approximately 30% prevalence of active HCV infection and despite the young cohort age, 25% had ≥F2 fibrosis. About two-thirds had ongoing alcohol use, and not unsurprisingly, this was a predictor of cirrhosis, highlighting that in PEH, besides HCV, additional risk factors need addressing. Despite considerable comorbidity and competing health priorities, our linkage to care was excellent with >90% of those eligible receiving DAA treatment. Our high SVR12 rates of >80% are a testament to the successful delivery of such a service. Another novel aspect of our work was reporting the significant improvement in both generic and liver specific HRQoL after HCV cure. Finally, we showed that detecting and curing HCV in the community could be achieved at modest costs of just over £600 (British pound 2022) per person. Our model in fact has been adopted by two of the largest HCV centres in England^22^ and has been endorsed by the European Centre for Disease Control and Prevention as a model of good practice.^23^

Currently only about 11 high income countries globally are on track to achieve HCV elimination.^24^ PEH remains an important group that needs attention. A recent meta-analysis assessing the care cascade in the DAA era reported a 66% linkage to care in the homeless population, with cure rates of just 17%.^25^ Our high linkage to care and SVR12 rates reflect the decentralised and integrated service with novel innovative strategies including home delivery of DAAs, point of care (POC) testing as well contingency management. An earlier systematic review showed that POC RNA testing reduced time to treatment initiation more than three-fold (19 days *vs.* 64 days) with significantly higher treatment uptake (77%–81% *vs.* 53%). Same site services outperformed others, reinforcing the need for integrated care.^26^ While contingency management remains controversial amongst PEH,^27^ in our opinion, the food vouchers positively impacted engagement. About 60% of our cohort had support from peer mentors, another factor associated with higher linkage to care,^28^ though not necessarily SVR12 as shown in this study and by others.^28^ HCV cure rates in PEH range between 82%–92%,^2^ which is consistent with our study. As expected, virological failures accounted for only 15% of all the non-SVR12, our mITT SVR12 rates being almost 100%, consistent with that observed in clinical trials.^29^ We found self-reported treatment adherence ≥80% to be the only independent predictor of SVR12, and this is corroborated by recently published literature.^30^ However, unlike Beiser *et al.*, we did not find homelessness and recent IDU to predict SVR12.^31^

Both homelessness and having HCV infection can adversely affect HRQoL.^32^^,^^33^ This is one of the first studies to show the impact of SVR12 on HRQoL amongst PEH. Despite the considerable comorbidity, we found significant improvements post SVR12 in generic domains (General and Mental Health, Social Functioning) as well as liver specific domains such as Symptoms and Effects of Liver Disease, Distress and Loneliness. However, the long-term impact of SVR12 on HRQoL remains unclear. A recent Canadian study showed that while health utilities after hospital-based HCV treatment steadily improved, utilities for the community-based cohort improved between baseline and 12-weeks post-treatment, but then decreased. This may be attributed to comorbid health and social conditions that are not meaningfully addressed by HCV treatment.^34^ This confirms the need to think beyond just HCV cure in this cohort. Some studies have in fact shown antiviral therapy to be associated with a significant reduction in injection frequency, risk practices, and homelessness,^35^ though these were not assessed in the current study.

We showed a high screening reach, treatment success rates, low drop out, and modest costs indicating that this model of case finding, and treatment is effective and efficient. Use of community POC HCV testing was found to be the most dominant approach to cost-effectiveness during the national Egyptian HCV elimination programme.^36^ This contrasts with opportunistic testing and referring PEH to specialist hospital clinics.^7^ The cost per case detected in the current study was higher and the cost per cure was lower than in our previous single-site study based at an Addiction Centre.^6^ This reflects differences in the screening and treatment protocols and unit costs. Screening costs can be kept lower by not using venous blood samples. Similarly, it could be argued that genotyping is futile in the era of pangenotypic regimens, but this reflected current national standard practice.

Our study did have some limitations. Adherence was self-reported, so could have been inaccurate. Additionally, a variety of tests were used for BBV screening, however, this reflected real-world practice. Our findings on costs may not be applicable nationally or internationally but are still helpful in providing an estimate for healthcare professionals setting up such a service. Peer mentor input was inconsistently recorded and was not included in our costs analysis but merits further exploration for its impact on screening uptake, treatment adherence, SVR12, and costs. In addition, only about half of the cohort was retested for re-infection, which might explain why our re-infection rates were lower than those reported in the literature,^37^ although they were consistent with national data.^4^ Finally, less than 50% of the cohort completed the HRQoL questionnaires, but despite this, significant improvements were seen in general and liver specific domains.

In conclusion, the END C study has shown that despite the significant burden from HCV and alcohol in PEH, a multisite regional decentralised service is effective in linking one of the most vulnerable cohorts with HCV infection into care. Our high SVR12 rates support the novel linkage to care strategies used in this study. Additionally, despite considerable comorbidity, at least in the short term, successful HCV cure can result in significant improvement in both generic and liver specific HRQoL. We have also demonstrated that this can be achieved at modest costs. Such models of care are paramount if we are to achieve global HCV elimination.

## Abbreviations

BBV, blood borne virus; CBT, capillary blood test; DAA, direct acting antiviral; DBST, direct blood spot test; HBcAb; hepatitis B core antibody; HBsAg, hepatitis B surface antigen; HCC, hepatocellular cancer; HRQoL, health-related quality of life; IDU, injecting drug use; ITT, intention to treat; LLD, lower limit of detection; LSM, liver stiffness measurement; MDM, multidisciplinary meeting; MHCS, mental health composite score; NHSE, National Health Service England; OAT, opioid agonist treatment; ODN, Operational Delivery Network; PROM, patient reported outcome measure; PEH, people experiencing homelessness; PCR, polymerase chain reaction; PHCS, physical health composite score; POC, point of care; PROMs, patient reported outcome measures; PWUD, people who use drugs; SFLDQoL, short from liver disease quality of life; SVR, sustained virological response; TE, transient elastography.

## Ethical approval

Ethical approval for the study was obtained (Brighton and Sussex Medical School Research Governance Ethics Committee (ER/BSMS1398/1), with all participants signing an informed consent form.

## Financial support

The END C study is predominantly funded by a research grant from Gilead Sciences (CHIME stream ISR-GB-18-10442) with a small contribution from the Sussex Operational Delivery Network. The funders were not involved in the study design, data collection/analysis and manuscript preparation.

## Conflicts of interest

SV research grants and consultancy Gilead Sciences; speaker fees Dr Falk; receipt of long-term abdominal drains from Rocket Medical plc and Becton Dickinson; AM, RM, EG, AMBJ, HG, LS, MOS, VC, NS, TB none.

## Authors’ contributions

AM recruitment, data collection and statistical analysis; RM, EG, LS, MOS, VC, NS recruitment and data collection; AMBJ statistical analysis oversight and PRO analysis; HG and TB health economic analysis; SV, AM, AMBJ and HG wrote the first draft of the manuscript. All co-authors contributed to and approved the final draft. SV is study guarantor.

## Data availability statement

The data that support the findings of this study are available upon reasonable request from the corresponding author.
