# Supplementary material for: A novel multisite model to facilitate hepatitis C virus elimination in people experiencing homelessness
Source: JHEP Rep. 2024 Aug 12;6(11):101183. doi: 10.1016/j.jhepr.2024.101183 (PMC11546132; doi:10.1016/j.jhepr.2024.101183)
Supplement: Multimedia component 1 [file mmc1.pdf]

A novel multisite model to facilitate hepatitis C virus elimination in people experiencing homelessness

Adele Mourad, Rona McGeer, Emma Gray, Anna-Marie Bibby-Jones, Heather Gage, Lidia Salvaggio, Vikki Charles, Natasha Sanderson, Margaret O'Sullivan, Thomas Bird, Sumita Verma

Table of contents

Supplementary file 1……………………………………………………………….………...2

Fig. S1………………………………………………………………………….………...4

Table S1…………………………………………………………………………………….5

Table S2…………………………………………………………………………………….6

Table S3…………………………………………………………………………………….7

Table S4…………………………………………………………………………………….9

Table S5………………………………………………………………………………….....11

Table S6………………………………………………………………………………….....13

Supplementary File 1. Analysis of questionnaires

Reprinted with permission John Wiley and Sons (Sullivan M, et al. ITTREAT (Integrated Community Test - Stage - TREAT) Hepatitis C service for people who use drugs: Real-world outcomes. Liver Internat. 2020;40:1021-1031)

Short Form Liver Disease Quality of Life (SFLDQoL)

A 25-item questionnaire, subtotalled into nine domains: distress, stigma, memory, symptoms, sleep, hopelessness, effect of liver disease, loneliness and sex, scored largely on a 6-point Likert scale. After data entry depending on the mechanism of missing data a suitable imputation technique was selected and sensitivity analysis carried out. Each item was transformed to a continuous variable (0-10 scale using an algorithm provided by the authors) and combined to form the following subscales: symptoms of liver disease, effects of liver disease, memory/ concentration, sleep, hopelessness, distress, loneliness, stigma of liver disease and sexual functioning problems. These were combined to form a total SFLDQOL summary score. As above, a descriptive summary of the outcome was carried out at each time point and the absolute change in the score. Bivariate analysis was performed to explore the relationship between the change in total SFLQOL score and the explanatory variables (patient characteristics) using appropriate methodology depending on the type of data: Kendall's tau for continuous or ordinal explanatory variables (assuming non-normal distribution of the outcome variable/total SFLQOL score); Pearson's correlation for normally distributed continuous variables. Multiple regression was used to explore which correlating factors significantly explained the variation in the outcome after treatment.

Short form -12 v2 (SF-12 v2)

Has 12 items which are transformed into the following domains on a scale of 0-100 where higher scores indicate better health: role physical, general health, vitality, physical functioning, role emotional, social functioning, bodily pain, mental health, physical health composite (PCS) score and mental health composite score (MCS).

Computer-based scoring of the SF-12v2 (Optum's scoring software v4.5), involved (a) recoding item response values, (b) summing recoded response values for all items in a given scale to obtain the scale raw score, (c) transforming the scale raw score to a 0–100 score, (d) transforming the 0–100 score to a z score, and (e) transforming the scale z score to a T score (mean = 50, standard deviation = 10). The PCS score was computed by: (a) multiplying each health domain z score by a scale-specific physical factor score coefficient, (b) summing the resulting

products, and (c) converting the product total to a T score. The MCS score was computed in the same manner, instead using scale-specific mental factor score coefficients. Explorations of the change in the SF-12v2 and contributing factors was carried out in the same way as for SFLDQO

EQ-5D-5L:

A five item composite profile score (16) (mobility, self-care, usual activities, pain/discomfort, and anxiety/depression), each scored on a 5 point scale (no problem to severe problem/unable), and converted to an index value range (100 (best) to -0.59) which can be used for the purpose of calculating quality adjusted life years (QALYs), as recommended by National Institute for Health and Care Excellence for cost-effectiveness analysis (17); and a 20 cm vertical Visual Analogue Scale (VAS) for self-rated health status with range 0 (worst imaginable) to 100 (best).

Fig. S1. Sussex Operational Delivery Network (ODN) map showing sites where END C homeless service was provided

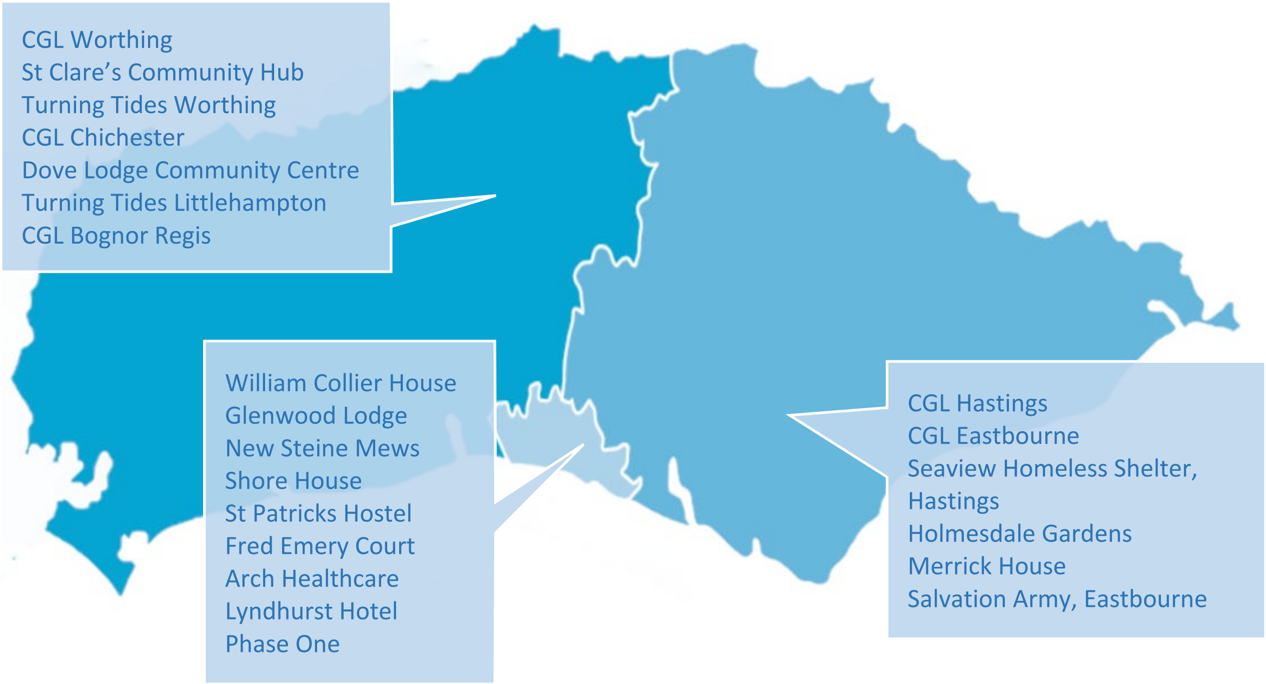

Table S1. Univariate and multivariate analysis of predictors of a positive HCV PCR

	Univariate analysis			Multivariate analysis		
**Key variables**	OR	CI	P value	OR	95% CI	P value
Age (per year increase in age)	0.99	0.97-1.01	0.288			
Male	1.02	0.61-1.71	0.930			
IDU (current/past)	31.30	14.05-69.75	<0.001	14.46	5.75-36.39	<0.001
Current IDU	5.98	3.68-9.70	<0.001			
Current non-IDU	2.39	1.48-3.85	<0.001			
Alcohol use (current/past)	0.46	0.26-0.81	0.007			
Currently drinking alcohol	0.50	0.32-0.78	0.002			
Receiving OAT	8.99	5.53-14.61	<0.001	2.39	1.35-4.23	0.003
Drug overdose	2.50	1.61-3.88	<0.001			
Ever incarcerated	2.65	1.70-4.13	<0.001			
Homeless at initial assessment	0.67	0.42-1.08	0.099	0.45	0.23-0.86	0.015
Any psychiatric diagnosis	2.17	1.22-3.86	0.021			
Cirrhosis (F4 Vs F0-F3)	2.36	1.22-4.57	0.011			
Shared paraphernalia ever	9.75	5.85-16.25	<0.001	2.20	1.18-4.09	0.013

IDU Injecting drug use; OAT opioid agonist treatment

Logistic regression analysis was used to model the relationship between the binary dependent outcomes (0 vs.1) HCV RNA positive (yes/no) and key independent factors. A multivariate logistic regression model was then derived to look at the relationship between the key factors and the dependent outcome. To build the model, the statistically significant key factors from bivariate analysis were added to the null model using forward selection, where the factor with the highest significant p-value ($p < 0.05$), based on the likelihood ratio test, was added next. Factors were removed from the model if $p \geq 0.05$. Only statistically significant variables on multivariate analysis are reported.

Table S2 Univariate and multivariate analysis of predictors of cirrhosis

	Univariate analysis			Multivariate analysis		
**Key variables**	OR	CI	P value	OR	CI	P value
Age (per year increase in age)	1.02	0.99-1.06	0.170			
Male	0.97	0.44-2.14	0.942			
IDU (current/past)	1.44	0.74-2.79	0.280			
Current IDU	1.00	0.48-2.10	0.990			
Current non-IDU	0.71	0.36-1.37	0.303			
Alcohol use (current/past)	1.98	0.58-6.72	0.272			
Currently drinking alcohol	2.79	1.14-6.85	0.025	1.01	1.00-1.01	<0.001
Receiving OAT	2.04	1.05-3.96	0.034			
Drug overdose	1.05	0.54-2.04	0.883			
Ever incarcerated	1.24	0.64-2.38	0.520			
Homeless at initial assessment	0.60	0.30-1.18	0.138			
Any psychiatric diagnosis	1.75	0.71-4.34	0.225			
HCV PCR Positive	2.36	1.22-4.57	0.011	4.02	1.89-8.57	<0.001

IDU injecting drug use; OAT opioid agonist treatment

Logistic regression analysis was used to model the relationship between the binary dependent outcomes (0 vs.1), cirrhosis (yes/no) and key independent factors. A multivariate logistic regression model was then derived to look at the relationship between the key factors and the dependent outcome. To build the model, the statistically significant key factors from bivariate analysis were added to the null model using forward selection, where the factor with the highest significant p-value ($p < 0.05$), based on the likelihood ratio test, was added next. Factors were removed from the model if $p \geq 0.05$. Only statistically significant variables on multivariate analysis are reported.

Table S3. Descriptive statistics for SF-12v2 & SFLDQoL scores prior to and at end of HCV treatment corresponding to Figs c and 1d

	Observed scores						Pre-post change in scores				
	Pre			Post					Lower	Upper	
SF-12v2 Outcome	N	Mean	SD	N	Mean	SD	Estimate	SE	95% CI	95% CI	p-value
Physical Functioning	38	36.1	11.9	38	40	12.7	3.8	2.1	-0.2	7.9	0.062
Role Physical	39	38	10.5	38	39.8	11.1	1.8	1.9	-2.0	5.6	0.35
Bodily Pain	38	35.5	13.5	39	39.2	13.5	3.5	2.3	-1.0	7.9	0.125
General Health	39	30.8	10.2	39	36.8	11.5	6.1	2.1	1.9	10.2	0.004
Vitality	39	39.2	10.2	39	42.8	10.5	3.6	2.1	-0.5	7.8	0.088
Social Functioning	39	33.3	12.7	39	37.4	12.2	4.1	2.1	0.0	8.3	0.049
Role Emotional	38	30.3	12.4	38	33.0	12.7	2.6	2.0	-1.2	6.5	0.177
Mental Health	39	35.9	9.7	39	39.8	9.6	3.9	1.8	0.4	7.4	0.028
Physical Health Composite Score	37	37.4	10.6	38	40.7	12.2	3.4	1.9	-0.3	7.2	0.069
Mental Health Composite Score	37	33.5	9.8	38	37.4	9.3	3.8	1.8	0.3	7.2	0.034

	Observed scores						Pre-post change in scores				
	Pre			Post					Lower	Upper	
SFLDQoL Outcome	N	Mean	SD	N	Mean	SD	Estimate	SE	95% CI	95% CI	p-value
Symptoms	39	51.8	21.7	39	66.6	22.6	14.8	3.4	8.1	21.4	<0.001
Effect	35	53.9	32.2	33	76.6	22.7	24.2	5.0	14.3	34.1	<0.001
Memory	37	42.4	26.5	39	50.6	23.9	6.4	3.7	-0.9	13.7	0.086
Distress	38	53.3	32.1	37	69.9	29.4	16.7	5.6	5.7	27.7	0.003
Sleep	38	47.1	19.4	39	53.3	17.2	5.9	3.2	-0.3	12.2	0.062
Loneliness	38	59.9	22.9	39	70.3	20.5	10.4	3.6	3.3	17.5	0.004
Hopelessness	38	55	24.2	39	60.8	22.4	6.0	3.8	-1.3	13.4	0.109
Stigma	37	59.5	26.6	37	67.5	26.5	7.6	5.1	-2.3	17.5	0.132
Sex**	14	22.2	10.2	15	26.5	5.8	5.4	3.4	-1.3	12.1	N/A

N count; SD Standard Deviation; SE Standard Error; CI Confidence Interval;

P-value is paired Student’s t-test results comparing pre and post scores with 95% confidence intervals with bootstrapping (1000 reps) to deal with missing data. Missing data for the pre-post difference on the SFLDQoL Sex outcome was extensive (72%) so was excluded from statistical testing.

Table S4. Unit costs of tests (provided by local NHS laboratory)

	**Cost of tests (UK £,2022)**	**Initial screen**	**1st follow up for HCV PCR if HCV antibody is +ve**	**2nd follow up for genotype if HCV PCR +ve**
**Code**	**Description**	**Cost if HCV Antibody -ve**	**Cost if HCV antibody +ve, and PCR -ve**	**Cost if HCV antibody and PCR +ve**
	**Dry blood spot test (DBST)**			
	Hepatitis B surface antigen	4.32	4.32	4.32
	Hepatitis C antibody	4.16	4.16	4.16
	HIV antibody	6.21	6.21	6.21
	Hepatitis B core antibody	-	4.99	4.99
	If HCV antibody positive, then reflex qualitative HCV RNA	-	7.91	7.91
	Hepatitis C viral load done by CBT	-	48.59	48.59
	HCV genotype by CBT			20
	Collection kit *	6.73	6.73	6.73
	TOTAL	21.42	82.91	102.91
	**Capillary blood test (CBT)**			
HBSAG	Hep B surface antigen	4.58	4.58	4.58
HCA	Hepatitis C antibody	5.82	5.82	5.82
HIV12	HIV antibody	6.17	6.17	6.17
HCVL	Hep C Viral Load (reflex)	-	42.28	42.28
RELFEX	Hep C Genotype (reflex)	-	-	20.00
	TOTAL	16.57	58.85	78.85
	**Venous blood test**			
FBC	Full Blood Count	4.36	4.36	4.36
LFT	Liver Function Test	4.06	4.06	4.06
INR	International Normalised Ratio	4.36	4.36	4.36
	Hepatitis B surface antigen	4.65	4.65	4.65
	Hepatitis B core antibody	4.65	4.65	4.65
	HIV1and 2 antigen/antibody	4.65	4.65	4.65
	Hepatitis C antibody	8.20	8.20	8.20
	Hepatitis C Viral load (reflex)	-	69.41	69.41
	Hepatitis C Genotype		-	69.41
	TOTAL	34.93	104.34	173.75

Fibroscan	According to a recent report from the National Institute for Health and Care Excellence (NICE), there is no tariff for fibroScan but the company estimates that its use will cost between £50 and £400, depending on the Centre or whether the patient is being scanned for the first time or in follow up. (NICE: fibroScan for assessing liver fibrosis and cirrhosis in primary care, 2020). We have used the lowest value because, after allowing for inflation, this is consistent with another estimate in the literature which cites £43 at 2014-5 values (Srivastava A, Jong S, Gola A, Gailer R, Morgan S, Sennett K, et al. Cost-comparison analysis of FIB-4, ELF and fibroscan in community pathways for non-alcoholic fatty liver disease. BMC Gastroenterol. 2019;19:122)			
Abdominal Ultrasound	Abdominal ultrasound cost is from NIHR Interactive Costing Tool (iCT) Investigation and Intervention Tariff, Version 1.2, 14th April 2020 https://www.nihr.ac.uk/documents/interactive-costing-tool-ict-getting-started/12170 Accessed 21st May 2023			

*Collection kit comprises special collection card, lancet, steret, plaster, request card and pre-paid postal transport envelope + initial processing costs of dried blood spot samples (one charge per sample)

Table S5. Cost per case detected: Assumes blood borne virus screening done by DBST and CBT (no venous samples)

Test type	Total screened	Screening outcomes	n	Cost of screen (£)	Total[1] cost (£)*
DBST	223	Initial screen only (HCV antibody negative), includes 3 failed screens	167	21.42	3,577
		Follow up test if HCV antibody positive but HCV PCR negative	39	82.91	3,233
		Follow up test for genotype if HCV PCR positive (done using CBT)	17	102.91	1,749
		Total	*223*		*8,559*
CBT	36	Initial screen only (HCV antibody negative)	1	16.57	17
		Follow up test if HCV antibody positive but HCV PCR negative	9	58.85	530
		Follow up test for genotype if HCV PCR positive	26	78.85	2,050
		Total	*36*		*2,597*
CBT replacing venous	159	Initial screen only (HCV antibody negative), includes 1 failed screen	72	16.57	1,193
		Follow up test if HCV antibody positive but HCV PCR negative	14	58.85	824
		Follow up test for genotype if HCV PCR positive	73	78.85	5,756
		Total	*159*		*7,773*
**All**	**418**	**Total of DBST, CBT and Venous blood tests2**	**418**	**33**	**18,929**
		**+ Cost of nurse initial 30-minute consultation @ £33 per participant[3]**	**418**		**13,794**
		**GRAND TOTAL OF SCREENING COSTS**	**418**		**32,723**
**Number of cases detected: n=116 (n=17 by n=DBST; 99 by CBT)<br>COST PER CASE DETECTED: 32,723/ 116 = £282<br>COST PER CASE: 32,723/ 418 = £78**					

[1]Total costs are rounded to the closest £
[2] test costs are in Table S4
[3] Cost per hour, including oncosts and NHS facilities overheads of a Band 7 Nurse is £66 (21)

Cost per case detected was calculated as the total cost of all nurse time and tests performed, summed across all those invited to be screened, divided by the total number of individuals with a positive HCV RNA (a case), over the study period. Cost per screen was the total cost of all screens conducted divided by the number of people screened. Sensitivity analyses explored the impact of screening method on cost per case detected.

PCR polymerase chain reaction; DBST dry blood spot testing; CBT capillary blood

Table S6. Cost per cure: Assumes cost of fibroscan is £100 (not £50)

**Item**	**Number receiving**	**Unit cost (£)**	**Total cost (£)[1]**
FibroScan[2]	104	100	10,400
Abdominal ultrasound[2]	17	61	1,037
Contact with nurse Band 7; mean duration of contact = 129 minutes, at £1.10 per minute[3]	105	129 minutes at £66 per hour=£142	14,900
SVR12 blood by DBST	88	7.91	696
**Total cost**			**27,033**
**Cost per person achieving SVR12= 27,033/ 85 = £318**			

[1] Total cost are rounded to the closest £
[2] Test costs are in Table S4
[3] Cost per hour, including oncosts and NHS facilities overheads of a Band 7 Nurse is £66 (21)

To calculate the cost per cure of those receiving DAA, the total cost of tests and nurse time was summed across all individuals receiving treatment and divided by the total number of individuals achieving SVR12. Sensitivity analyses explored the impact of varying the cost of the fibroscan on cost per case cured

SVR12 sustained virological response; DBST dry blood spot test
